# MiR-200c-3p promotes ox-LDL-induced endothelial to mesenchymal transition in human umbilical vein endothelial cells through SMAD7/YAP pathway

**DOI:** 10.1186/s12576-021-00815-z

**Published:** 2021-09-15

**Authors:** Yongzhong Mao, Ling Jiang

**Affiliations:** 1 Department of Pediatric Surgery, Union Hospital, Tongji Medical College, Huazhong University of Science and Technology, 1277 Jiefang Avenue, Wuhan, 430022 Hubei China; 2 Department of Geriatrics, Union Hospital, Tongji Medical College, Huazhong University of Science and Technology, 1277 Jiefang Avenue, Wuhan, 430022 Hubei China

**Keywords:** miR-200c-3p, Ox-LDL, Endothelial to mesenchymal transition, SMAD7/YAP, Atherosclerosis

## Abstract

**Background:**

Endothelial to mesenchymal transition (EndMT) participates in the progression of atherosclerosis (AS). MiR-200c-3p has been implicated in EndMT. However, the functional role of miR-200c-3p in AS remains largely unknown. Here, we demonstrated the critical role of miR-200c-3p in regulating EndMT in AS.

**Methods:**

ApoE^−/−^ mice were fed with high-fat diet to establish AS mouse model, and human umbilical vein endothelial cells (HUVECs) were treated with oxidized low-density lipoprotein (ox-LDL) to mimic AS cell model. The expression of miR-200c-3p, SMAD7 and YAP in ApoE^−/−^ mice and HUVECs was detected by quantitative real-time PCR. Rhodamine phalloidin staining and Western blot were performed to observe cell morphology and EndMT marker expression of HUVECs. Luciferase reporter assay and Co-Immunoprecipitation were performed to verify the relationship among miR-200c-3p, SMAD7, and YAP.

**Results:**

MiR-200c-3p was highly expressed, and SMAD7 and YAP were down-regulated in the aortic tissues of ApoE^−/−^ mice and ox-LDL-treated HUVECs. MiR-200c-3p overexpression promoted the transformation of ox-LDL-treated HUVECs from cobblestone-like epithelial phenotype to a spindle-like mesenchymal phenotype. Meanwhile, miR-200c-3p up-regulation repressed the expression of endothelial markers CD31 and vWF and promoted the expression of mesenchymal markers α-SMA and vimentin in the ox-LDL-treated HUVECs. MiR-200c-3p inhibited SMAD7 and YAP expression by interacting with 3′ untranslated region of SMAD7. Moreover, miR-200c-3p promoted EndMT in ox-LDL-treated HUVECs by inhibiting SMAD7/YAP pathway.

**Conclusion:**

This work demonstrated that MiR-200c-3p promoted ox-LDL-induced EndMT in HUVECs through SMAD7/YAP pathway, which may be important for the onset of atherosclerosis.

**Supplementary Information:**

The online version contains supplementary material available at 10.1186/s12576-021-00815-z.

## Background

Atherosclerosis (AS) is the most common disease of the cardiovascular system. Coronary heart disease and stroke caused by AS have become one of the important causes of death in the world [1]. AS is a pathological process based on vascular endothelial cell damage and characterized by lipid infiltration and inflammation of the blood vessel wall [2]. However, the pathogenesis of AS has not been fully elucidated. Thus, how to prevent and treat AS more effectively has become an urgent problem.

Endothelial to mesenchymal transition (EndMT) is the process of endothelial cells transforming into mesenchymal cells under the action of various stimulating factors [3]. In this process, endothelial cells gradually lose their morphology and function, and at the same time acquire the phenotypes and characteristics of mesenchymal cells, such as proliferation, migration, and collagen synthesis ability [4]. Accumulation studies have confirmed that EndMT plays a crucial role in all stages of AS [5, 6]. The extent of EndMT is closely associated with instability of the plaque in AS [7]. High-glucose and oxidized low-density lipoprotein (ox-LDL) induce EndMT in human aortic endothelial cells [8, 9]. Advanced glycation end products promote EndMT of human endothelial cells and damage the polarization of endothelial cells through AKT2 signaling cascades [10]. Thus, regulating EndMT may be an important target for the prevention and treatment of AS.

MicroRNA (miRNA) is a family of small non-coding RNAs that can interfere with transcription and post-transcriptional processes. The study of Magenta et al. has confirmed that oxidative stress enhances the expression of miR-200c in human umbilical vein endothelial cells (HUVECs), and inhibition of miR-200c promotes cell proliferation and inhibits apoptosis in H_2_O_2_-treated HUVECs [11]. Further study has shown that miR-200c is related to atherosclerotic plaque instability in carotid arteries, indicating that miR-200c may be an biomarker for atherosclerotic plaque progression [12]. Thus, miR-200c participates in the progression of AS. MiR-200c-3p is a member of the miR-200c gene family. Predictive analysis (Targetscan; http://www.targetscan.org/vert_71/) of miR-200c-3p revealed that there were binding sites between miR-200c-3p and 3′ untranslated region (UTR) of Smad family member 7 (SMAD7). A previous study has showed that SMAD7 and yes-associated protein (YAP) can interact with each other [13]. Moreover, SMAD7 has been reported to participate in various diseases through regulating the EndMT. In the achilles tendon injury rat model, SMAD7 prevents heterotopic ossification via inhibiting EndMT [14]. Aspirin-Triggered Resolvin D1 inhibits TGF-β1-induced EndMT in HUVECs by up-regulating SMAD7 expression [15]. Recently, studies have revealed that YAP plays an important role in different biological processes in endothelial cells, including angiogenesis and AS [16, 17]. YAP up-regulation in myeloid cells aggravates atherosclerotic lesion size and promotes macrophage infiltration, whereas inhibition of YAP reduces atherosclerotic plaque [18]. Naringin inhibits apoptosis, EndMT, and inflammatory response in ox-LDL-treated HUVEC by regulating the YAP pathway [19, 20]. YAP also has been confirmed to induce EndMT in oral submucous fibrosis [21]. Therefore, we speculated that miR-200c-3p may induce EndMT through by regulating the SMAD7/YAP pathway, thereby participating in the progression of AS.

## Materials and methods

### Animals

Male C57BL/6 mice (8 weeks old, weighting 15–20 g) and ApoE^−/−^ mice (8 weeks old, weighting 15–20 g) with the same genetic background were obtained from Beijing Vital River Laboratory Animal Technology Co., Ltd. (Beijing, China). These mice were housed under SPF conditions with 12 h light–dark cycle. ApoE^−/−^ mice (n = 6) were fed with high-fat diet (HFD) containing 21% fat and 1.25% cholesterol for 16 weeks to construct AS mouse model. C57BL/6 mice (n = 6) were fed with normal diet and served as control. All protocols were carried out in accordance with the Guide for the Care and Use of Laboratory Animals (National Institutes of Health) and authorized by the Ethics Committee of Tongji Medical College, Huazhong University of Science and Technology.

After modeling, the mice were euthanized by cervical dislocation. The aortic tissues of mice were separated and used for quantitative real-time PCR (qRT-PCR) analysis.

### Cell culture

HUVECs were obtained from Cell Bank of the Chinese Academy of Sciences (Shanghai, China). HUVECs were cultured in Dulbecco’s modified Eagle medium (DMEM, Gibco, Gaithersburg, MD, USA) supplemented with 10% fetal bovine serum (FBS, Gibco) and 1% penicillin/streptomycin (Solarbio, Beijing, China) at 37 °C and 5% CO_2_. HUVECs were treated with different concentrations (0, 10, 20, 40 μg/mL) of ox-LDL (Solarbio) for 48 h.

### Cell transfection

For SMAD7 or YAP overexpression, the vector pcDNA3.1 carrying full length of SMAD7 (pcDNA3.1-SMAD7) or YAP (pcDNA3.1-YAP) was constructed by GeneChem (Shanghai, China). The empty vector pcDNA3.1-NC served as negative control (NC). MiR-200c-3p mimic (5′-UAAUACUGCCGGGUAAUGAUGGA-3′), miR-200c-3p inhibitor (5′-UCCAUCAUUACCCGGCAGUAUUA-3′), mimic NC (5′-UUGUACUACACAAAAGUACUG-3′), and inhibitor NC (5′-CAGUACUUUUGUGUAGUACAA-3′) were synthesized by GeneChem. HUVECs were transfected with the plasmids using Lipofectamine 2000 Transfection Reagent (Invitrogen, Carlsbad, CA, USA). After 48 h of transfection, the transfected HUVECs were collected and treated with 40 μg/mL ox-LDL for 48 h.

### Gene expression

The expression of genes in HUVECs and aortic tissues of mice was examined by qRT-PCR. TRIzol reagent (Invitrogen) was used to extract total RNA from HUVECs or aortic tissues. RNA integrity was examined by 1.5% agarose gel electrophoresis. The reverse transcription of cDNA was carried out applying RevertAid First Strand cDNA Synthesis Kit (Thermo Fisher Scientific, Waltham, MA, USA). For analysis of miRNA expression, cDNA was generated applying the same kit with stem-looped miRNA-specific reverse transcription primers. The relative expression of mRNA and miRNA was examined by performing qRT-PCR using TB Green® Premix Ex Taq™ II (Takara, Tokyo, Japan) on applied Biosystems 7300 Real-Time PCR system (Applied Biosystems, Foster City, CA, USA). The relative expression of miRNA was normalized by U6, and the relative expression of mRNA was normalized by GAPDH. Data were analyzed using 2^−∆∆CT^ method. Below are the primers used for qRT-PCR.

**Table:** 

Gene	Primer sequence (5′–3′)
Primers used for qRT-PCR	
miR-200c-3p-forward	AGGGCTAATACTGCCGGGTAA
miR-200c-3p-reverse	CAGTGCAGGGTCCGAGGTAT
SMAD7-forward	GCTATTCCAGAAGATGCTGTTC
SMAD7-reverse	GTTGCTGAGCTGTTCTGATTTG
YAP-forward	CATGTTCAAAGGCTTGGGTGG
YAP-reverse	TCACGGTTCCAGTCTAGGCA
U6-forward	GCTTCGGCAGCACTAATACTAAAAT
U6-reverse	CGCTTCACGAATTTGCGTGTCAT
GAPDH-forward	GGAGTCAACGGATTTGGTC
GAPDH-reverse	TGGGTGGAATCATATTGGAACAT

### Protein expression

Western blot (WB) was performed to estimate the expression of proteins in HUVECs. Total protein was extracted from HUVECs using Total Protein Extraction Kit (Solarbio). BCA Protein Assay Kit (Solarbio) was used to assess the concentration of proteins. Protein samples (25 μg) were separated by 10% sodium dodecyl sulfate polyacrylamide gel electrophoresis and transferred onto a nitrocellulose membrane (Thermo Fisher Scientific). The membranes were blocked with 5% skim milk at room temperature for 2 h, and then incubated with the primary antibodies, SMAD7 (1:1000; Proteintech, Wuhan, China), YAP (1:2000; Proteintech), CD31 (1:1000; Proteintech), vWF (1:1000; Proteintech), and α-SMA (1:1000; Proteintech) at 4 °C for 12 h. After washing with Tris-Buffered saline Tween, the membranes were incubated with horseradish peroxidase-conjugated secondary antibody (1:5000; Proteintech) at room temperature for 1 h. β-actin antibody (1:5000; Proteintech) was used as a reference protein for normalization. The data were analyzed by Image J software (National Institutes of Health, Bethesda, MD, USA).

### Cell morphology analysis

Rhodamine phalloidin staining was performed to observe cell morphology of HUVECs. After 24 h of culture, HUVECs suspension was fixed with 4% formaldehyde at 4 °C for 20 min. For membrane permeabilization, HUVECs suspension was treated with 0.3% Triton X-100 for 20 min. Subsequently, HUVECs suspension was stained with FITC-rhodamine phalloidin (Thermo Fisher Scientific) at room temperature for 1 h. DAPI Fluoromount-G® (Southern Biotech, Birmingham, USA) was used to stain nuclei for 15 min in dark. Finally, the cell morphology was observed under a confocal laser scanning microscopy (ZEISS, Oberkochen, Germany).

### Luciferase reporter assay

Luciferase reporter assay was performed to verify the interaction between miR-200c-3p and SMAD7. 3′ UTR of SMAD7 containing the predicted miR-200c-3p binding sites was cloned into pmir-GLO vectors, generating the vectors pmir-GLO-SMAD7-Wt (wild type) and pmir-GLO-SMAD7-Mut (mutant type) (GeneChem). The Wt (Mut) SMAD7 vector and miR-200c-3p mimic or mimic NC were co-transfected into 293 cells (Cell Bank of the Chinese Academy of Sciences, Shanghai, China) by using Lipofectamine 2000 Transfection Reagent. The transfected 293 cells were cultured in DMEM containing 10% FBS and 1% penicillin/streptomycin at 37 °C and 5% CO_2_ for 48 h. After that, Dual luciferase assay kit (Promega, Madison, USA) was used to measure the activities of luciferase on luciferase assay system (Ambion, Austin, TX, USA).

### Co-Immunoprecipitation

Co-Immunoprecipitation (Co-IP) assay was performed to determine the interaction between SMAD7 and YAP in HUVECs. After 24 h of culture, HUVECs were collected and washed with PBS. Then, HUVECs were lysed with RIPA Lysis Buffer (Beyotime, Shanghai, China) at 4 °C for 30 min. The cell lysates were harvested by centrifugation at 13,000 g for 15 min. A portion of the cell lysates were removed as Input for WB. Subsequently, the cell lysates were incubated with the primary antibody, SMAD7 (1:50; Thermo Fisher Scientific) at 4 °C for 3 h. Furthermore, the cell lysates were incubated with Protein A/G Plus-Agarose (Santa Cruz Biotechnology, Santa Cruz, CA, USA) at 4 °C for 6 h. Immunoprecipitates were collected and washed with pre-cooled PBS for several times. After that, the immunoprecipitates and a portion of the cell lysates in Input group were analyzed by WB assay using the primary antibodies, SMAD7 (1:1000; Proteintech) and YAP (1:2000; Proteintech).

### Statistical analysis

Each assay was repeated for 3 times. Data were analyzed statistically using GraphPad Prism 6.0 Software (GraphPad Inc., San Diego, CA, USA) and are expressed as mean ± standard deviation. Statistical significance was examined using Two-tailed Student’s *t* test or one-way ANOVA. Differences were defined as statistically significant if *P* < 0.05.

## Results

### *MiR-200c-3p was up-regulated, SMAD7 and YAP were down-regulated in ApoE*^*−/−*^* mice.*

**Fig. 1:**
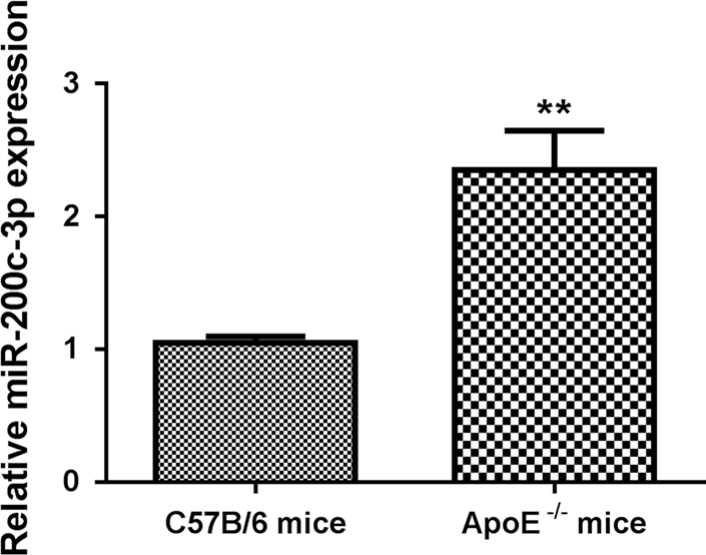
MiR-200c-3p was highly expressed in aortic tissues of ApoE^−/−^ mice. ApoE^−/−^ mice were fed with HFD to establish AS mouse model. Normally fed C57B/6 mice served as control. The expression of miR-200c-3p in aortic tissues of C57B/6 and ApoE^−/−^ mice was assessed by qRT-PCR. **P < 0.01 vs. C57B/6 mice group

We initially examined the expression of miR-200c-3p in aortic tissues of ApoE^−/−^ mice by qRT-PCR. Compared with normal C57B/6 mice, miR-200c-3p was highly expressed in aortic tissues of ApoE^−/−^ mice (Fig. 1). We also found that SMAD7 and YAP expressions were decreased in the aortic tissues of ApoE^−/−^ mice with respect to C57B/6 mice (Additional file 1: Figure S1A–C). Thus, miR-200c-3p, SMAD7, and YAP may be closely associated with AS development.

### MiR-200c-3p overexpression promoted EndMT in ox-LDL-treated HUVECs.

**Fig. 2:**
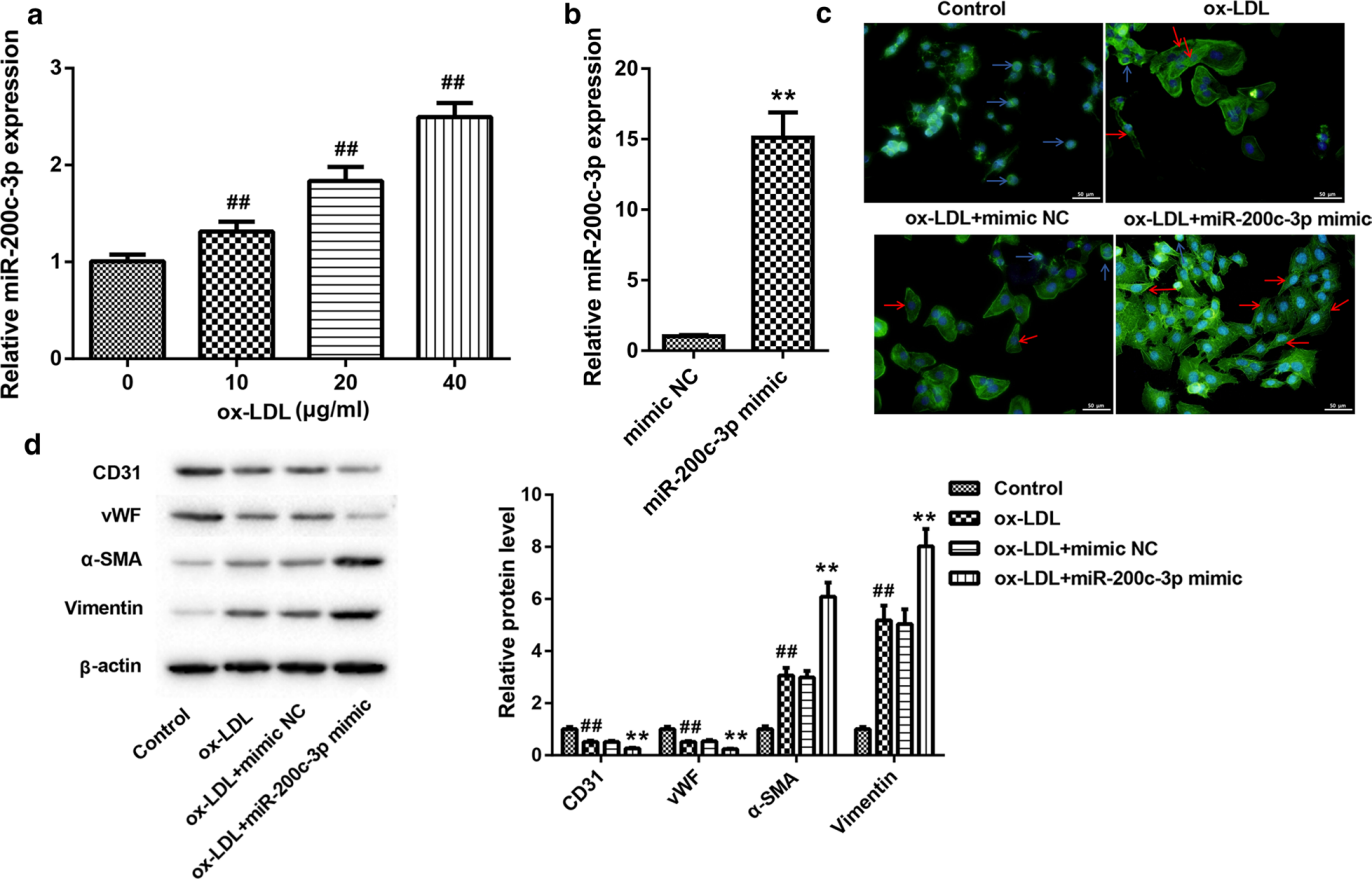
MiR-200c-3p overexpression promoted EndMT in ox-LDL-treated HUVECs. HUVECs were treated with ox-LDL at different concentrations (0, 10, 20, 40 μg/mL) for 48 h. **A** The expression of miR-200c-3p in HUVECs was assessed by qRT-PCR. **B** HUVECs were transfected with miR-200c-3p mimic or mimic NC. The expression of miR-200c-3p in the HUVECs was assessed by qRT-PCR. HUVECs were transfected with miR-200c-3p mimic or mimic NC, followed by 40 μg/mL ox-LDL treatment. **C** Rhodamine phalloidin staining was performed to observe cell morphology of HUVECs. The blue arrows indicate cobblestone-like cells, and the red arrows indicate the spindle-like cells. **D** WB was performed to estimate the expression of CD31, vWF, α-SMA, and vimentin in the HUVECs. **P < 0.01 vs. mimic NC or ox-LDL + mimic NC group; ^##^*P* < 0.01 vs. 0 or Control group

In order to determine the biological role of miR-200c-3p in AS, we treated HUVECs with ox-LDL to mimic AS cell model. The expression of miR-200c-3p was increased in the HUVECs after treated with different concentrations of ox-LDL, especially 40 μg/mL of ox-LDL (Fig. 2B). The mRNA and protein expressions of SMAD7 and YAP were reduced in HUVECs following ox-LDL treatment in a dose-dependent manner (Additional file 1: Figure S1D–F). Thus, ox-LDL at 40 μg/mL was used to treat HUVECs. Furthermore, we overexpressed miR-200c-3p in HUVECs. The data of qRT-PCR showed that the expression of miR-200c-3p was significantly enhanced in HUVECs in the presence of miR-200c-3p mimic (Fig. 2B). Moreover, we observed the influence of miR-200c-3p overexpression on cell morphology of HUVECs by rhodamine phalloidin staining. Normal HUVECs exhibited a cobblestone-like epithelial phenotype. The morphology of HUVECs changed significantly, displaying a spindle-like mesenchymal phenotype following ox-LDL treatment while losing their endothelial characteristic shape. However, miR-200c-3p overexpression further promoted the spindle-like mesenchymal phenotype of ox-LDL-treated HUVECs (Fig. 2C). The proportions of spindle-shaped mesenchymal cells were increased in HUVECs in the presence of ox-LDL. Treatment of ox-LDL-induced increase of spindle-shaped mesenchymal cell proportions was enhanced by miR-200c-3p overexpression (Additional file 1: Figure S2A). Next, we examined the expression of EndMT markers in HUVECs. WB data revealed that ox-LDL treatment repressed the expression of endothelial markers CD31 and vWF, whereas caused an up-regulation of mesenchymal markers α-SMA and vimentin in HUVECs. Overexpression of miR-200c-3p further repressed the expression of CD31 and vWF, and promoted the expression of α-SMA and vimentin in the ox-LDL-treated HUVECs (Fig. 2D). Therefore, miR-200c-3p overexpression promoted EndMT in ox-LDL-treated HUVECs.

### MiR-200c-3p inhibited SMAD7 and YAP expression by targeting SMAD7.

**Fig. 3:**
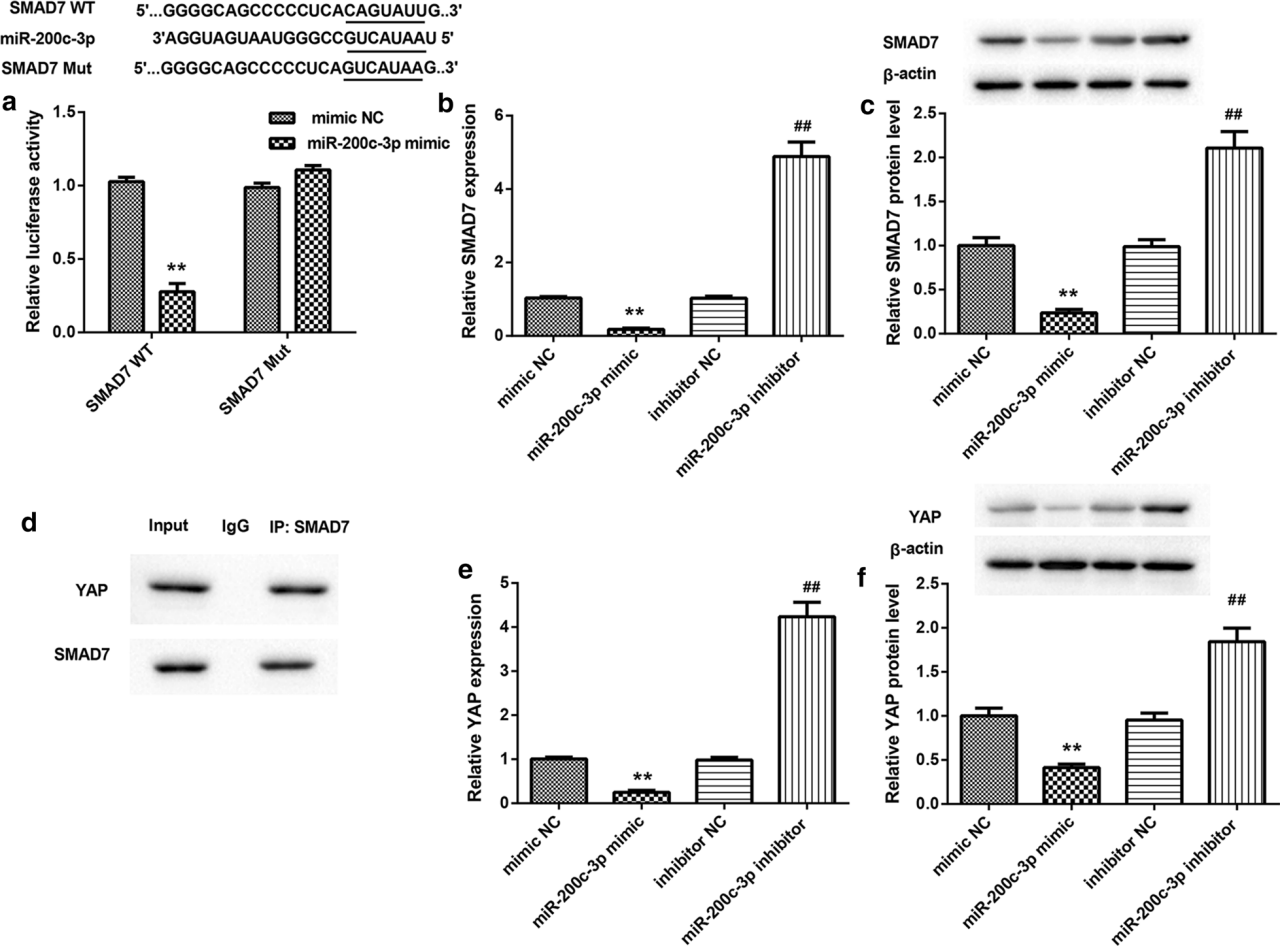
MiR-200c-3p inhibited SMAD7 and YAP expression. **A** Luciferase reporter assay was performed to verify the relationship between miR-200c-3p and 3′ UTR of SMAD7. The qRT-PCR (**B**) and WB (**C**) were performed to assess the expression of SMAD7 in the HUVECs following transfection of miR-200c-3p mimic, mimic NC, miR-200c-3p inhibitor, or inhibitor NC. **D** Co-IP assay was performed to determine the interaction between SMAD7 and YAP in HUVECs. The qRT-PCR (**E**) and WB (**F**) were performed to assess the expression of YAP in the HUVECs following transfection of miR-200c-3p mimic, mimic NC, miR-200c-3p inhibitor, or inhibitor NC. **P < 0.01 vs. mimic NC group; ^##^*P* < 0.01 vs. inhibitor NC group

Bioinformatics software analysis (Targetscan; http://www.targetscan.org/vert_71/) revealed that there were binding sites between miR-200c-3p and 3′ UTR of SMAD7. To verify this prediction, we performed luciferase reporter assay and found that miR-200c-3p interacted with 3′ UTR of SMAD7 (Fig. 3A). Then, we overexpressed or silenced miR-200c-3p in HUVECs, and examined the impact of miR-200c-3p on SMAD7 expression in HUVECs. As shown in Fig. 3B, C, miR-200c-3p overexpression inhibited SMAD7 gene and protein expression, while miR-200c-3p deficiency caused an up-regulation of SMAD7 in HUVECs. Moreover, the results of Co-IP assay confirmed that SMAD7 interacted with YAP in HUVECs (Fig. 3D). We also found that miR-200c-3p overexpression inhibited the mRNA and protein expression of YAP, whereas miR-200c-3p silencing enhanced the mRNA and protein expression of YAP in HUVECs (Fig. 3E and F). Thus, miR-200c-3p inhibited SMAD7 and YAP expression by targeting 3′ UTR of SMAD7.

### MiR-200c-3p promoted EndMT in ox-LDL-treated HUVECs by inhibiting SMAD7/YAP pathway.

**Fig. 4:**
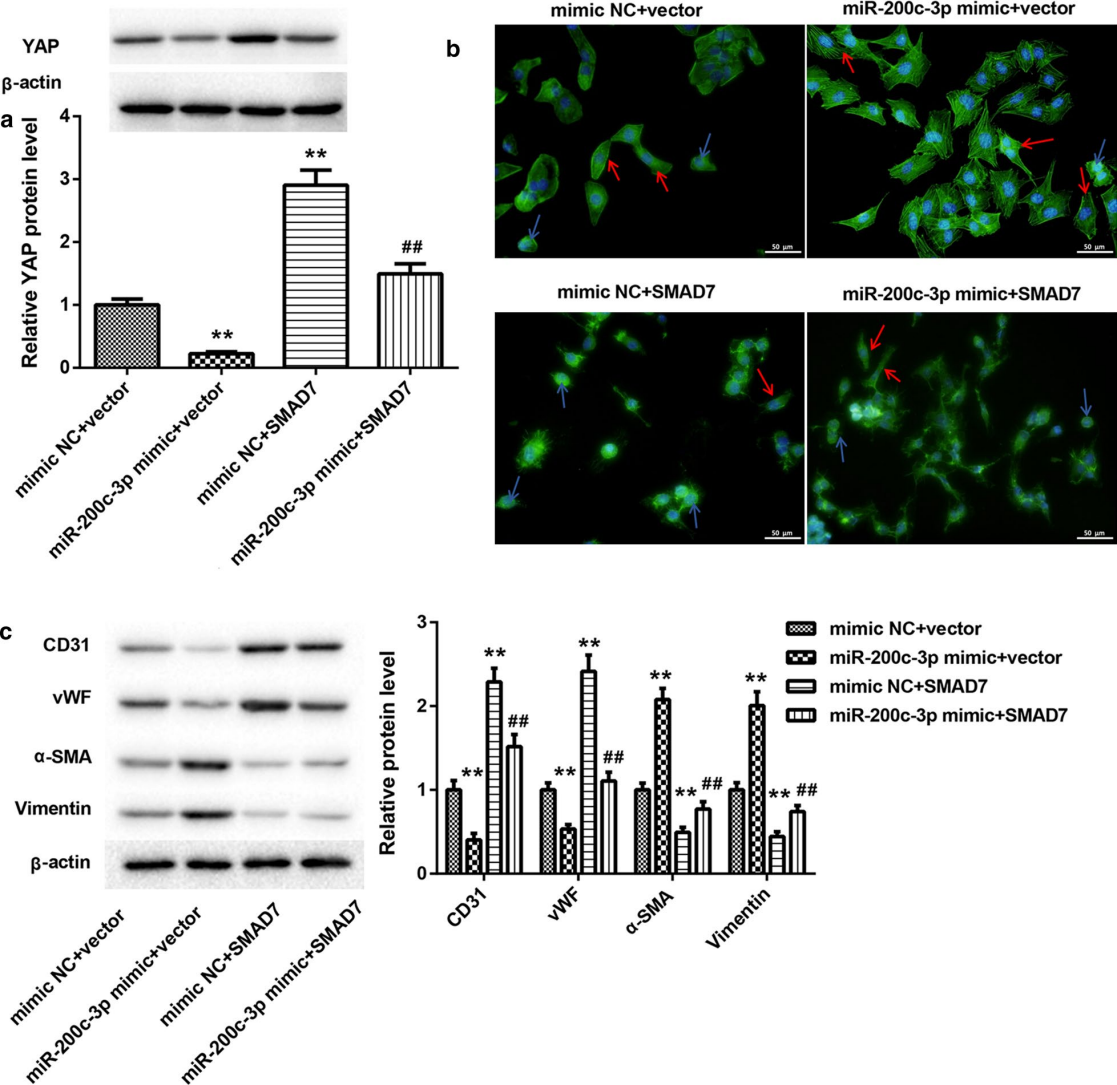
MiR-200c-3p promoted EndMT in ox-LDL-treated HUVECs by inhibiting SMAD7 expression. HUVECs were co-transfected with miR-200c-3p mimic or mimic NC and pcDNA3.1-SMAD7 or pcDNA3.1-NC, followed by 40 μg/mL ox-LDL treatment. **A** The expression of YAP in the HUVECs was assessed by WB. **B** Rhodamine phalloidin staining was performed to observe cell morphology of HUVECs. The blue arrows indicate cobblestone-like cells, and the red arrows indicate the spindle-like cells. **C** WB was performed to estimate the expression of CD31, vWF, α-SMA, and vimentin in the HUVECs. **P < 0.01 vs. mimic NC + Vector group; ^##^*P* < 0.01 vs. mimic NC + SMAD7 group

Finally, we determined whether miR-200c-3p can promote EndMT in HUVECs through SMAD7/YAP pathway. We overexpressed miR-200c-3p combined with SMAD7 in HUVECs. The results of WB showed that miR-200c-3p overexpression repressed YAP expression, whereas SMAD7 up-regulation led to up-regulation of YAP in the ox-LDL-treated HUVECs. Up-regulation of miR-200c-3p impaired SMAD7 overexpression-mediated promotion of YAP expression in the ox-LDL-treated HUVECs (Fig. 4A). Moreover, rhodamine phalloidin staining revealed that under ox-LDL treatment, HUVECs exhibited spindle-like mesenchymal appearance in the presence of miR-200c-3p mimic, while HUVECs displayed cobblestone-like endothelial phenotype following transfection with pcDNA3.1-SMAD7. The proportions of spindle-shaped mesenchymal cells in ox-LDL-treated HUVECs were increased by miR-200c-3p overexpression and suppressed by SMAD7 up-regulation. However, the influence conferred by SMAD7 overexpression was abolished by miR-200c-3p up-regulation (Fig. 4B and Additional file 1: Figure S2B). Furthermore, the WB analysis of EndMT markers showed that miR-200c-3p overexpression caused a down-regulation of CD31 and vWF, whereas led to an increase in the expression of α-SMA and vimentin in the ox-LDL-treated HUVECs. Overexpression of SMAD7 enhanced CD31 and vWF expression and inhibited α-SMA and vimentin expression in the ox-LDL-treated HUVECs, which was rescued by miR-200c-3p overexpression (Fig. 4C).

**Fig. 5:**
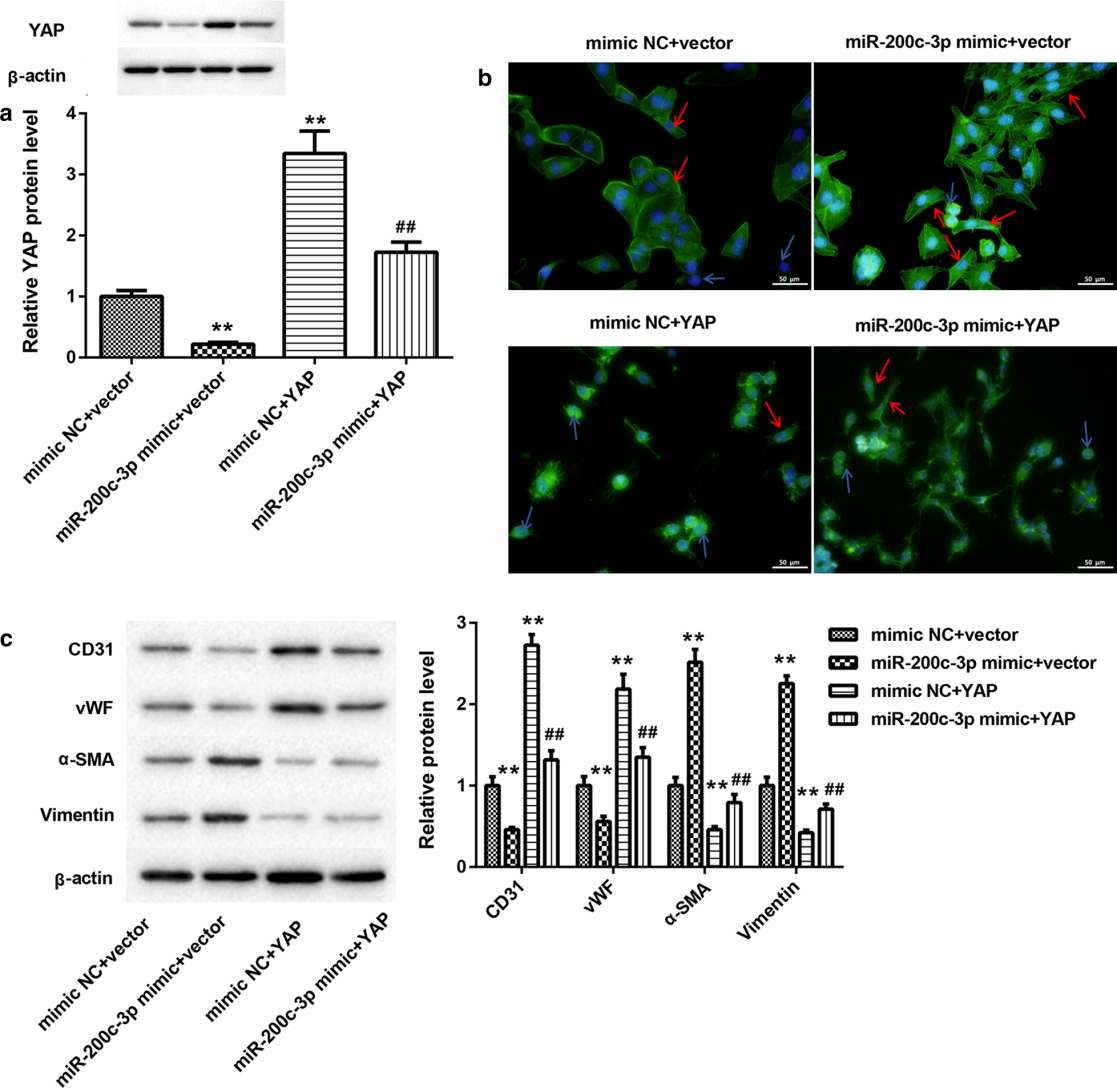
MiR-200c-3p promoted EndMT in ox-LDL-treated HUVECs by inhibiting YAP expression. HUVECs were co-transfected with miR-200c-3p mimic or mimic NC and pcDNA3.1-YAP or pcDNA3.1-NC, followed by 40 μg/mL ox-LDL treatment. **A** The expression of YAP in the HUVECs was assessed by WB. **B** Rhodamine phalloidin staining was performed to observe cell morphology of HUVECs. The blue arrows indicate cobblestone-like cells, and the red arrows indicate the spindle-like cells. **C** WB was performed to estimate the expression of CD31, vWF, α-SMA, and vimentin in the HUVECs. **P < 0.01 vs. mimic NC + Vector group; ^##^*P* < 0.01 vs. mimic NC + YAP group

In addition, HUVECs were co-transfected with miR-200c-3p mimic and pcDNA3.1-YAP. WB analysis revealed that miR-200c-3p overexpression significantly suppressed YAP expression in the ox-LDL-treated HUVECs. MiR-200c-3p up-regulation abolished YAP overexpression-mediated promotion of YAP expression in the ox-LDL-treated HUVECs (Fig. 5A). Then, we performed rhodamine phalloidin staining to observe the morphology of HUVECs. Most of ox-LDL-treated HUVECs exhibited spindle-like mesenchymal phenotype upon miR-200c-3p overexpression. YAP overexpression promoted the cobblestone-like endothelial appearance and reduced the proportions of spindle-shaped mesenchymal cells in ox-LDL-treated HUVECs, which was abolished by miR-200c-3p up-regulation (Fig. 5B and Additional file 1: Figure S2C). We also found that YAP up-regulation enhanced the expression of CD31 and vWF and inhibited the expression of α-SMA and vimentin in the ox-LDL-treated HUVECs. However, the influence of YAP up-regulation on CD31, vWF, α-SMA, and vimentin expression in ox-LDL-treated HUVECs was reversed by miR-200c-3p up-regulation (Fig. 5C).

Taken together, these data demonstrated that miR-200c-3p promoted EndMT in HUVECs by regulating SMAD7/YAP pathway.

## Discussion

MiR-200c-3p has been reported to participate in EndMT in various diseases. For example, the EndMT process that relies on miR-200 family is a reasonable biological mechanism that makes the bicuspid aortic valve more prone to aneurysms [22]. A previous study has confirmed that miR-200c-3p is highly expressed in human femoral arteries with atherosclerotic lesions [23]. The miR-200c-3p expression is also increased in the NaF-treated HUVECs, and NaF promotes apoptosis of HUVECs by regulating miR-200c-3p/Fas pathway [24]. These data suggest that miR-200c-3p is involved in the cardiovascular diseases. Consistently, our data found that miR-200c-3p expression was significantly enhanced in the ApoE^−/−^ mice and ox-LDL-treated HUVECs. Under the treatment of ox-LDL, the morphology of HUVECs changed from cobblestone-like endothelial appearance to spindle-like mesenchymal phenotype. Ox-LDL treatment also reduced the expression of endothelial markers CD31 and vWF, and enhanced the expression of mesenchymal markers α-SMA and vimentin in the HUVECs. MiR-200c-3p overexpression enhanced the proportions of spindle-shaped mesenchymal cells in ox-LDL-treated HUVECs. MiR-200c-3p overexpression accelerated the impact of ox-LDL-induced EndMT in HUVECs. EndMT is a process that is accompanied by the loss of cell polarity and intercellular adhesion, which leads to a spindle-shaped morphology with migratory and invasive phenotype [25]. During the EndMT, the expression of endothelial markers, such as CD31 and vWF, is decreased, whereas the expression of mesenchymal markers, such as α-SMA and vimentin, is enhanced [26]. Therefore, mesenchymal cells have myofibroblast-like contractile properties, enhanced proliferation and migration capabilities, and generate more extracellular matrix. At the same time, it also loses the functional characteristics of endothelial cells, such as anti-thrombosis ability and angiogenesis ability [25]. Therefore, this work showed that miR-200c-3p promoted ox-LDL-induced EndMT in HUVECs.

SMAD7 is known to have a crucial role in inflammatory response and fibrosis. Wei et al. have confirmed that SMAD7 is down-regulated, while the SMAD7 methylation is increased in the atherosclerotic plaques [27]. Homocysteine accelerates NF-κB-mediated vascular inflammation by enhancing the methylation levels of SMAD7 in human umbilical vein smooth muscle cells [28]. Thus, these data suggest that SMAD7 is associated with the progression of AS. In the present study, we found that SMAD7 and YAP were decreased in the ApoE^−/−^ mice and ox-LDL-treated HUVECs. MiR-200c-3p interacted with 3′ UTR of SMAD7 and repressed SMAD7 and YAP expression in HUVECs. SMAD7 overexpression promoted ox-LDL-treated HUVECs exhibited more cobblestone-like endothelial appearance, which was abolished by miR-200c-3p up-regulation. Thus, these data showed that overexpression of miR-200c-3p promoted EndMT in ox-LDL-treated HUVECs by suppressing the expression of SMAD7.

YAP has been reported to take part in the progress of EndMT. For example, YAP1 can promote the activation of key transcription factors required for EMT during the cardiac cushion development induced by TGFβ, such as Snail, Twist1, and Slug [29]. Arecoline-induced reactive oxygen species activate YAP and promote the initiation of EndMT, thereby leading to the occurrence of oral submucous fibrosis [21]. Salvianolic acid B exerts anti-atherosclerosis effects by inhibiting inflammatory response in ox-LDL-treated endothelial cells and pericytes via YAP/TAZ/JNK signaling pathway [30]. In the present study, we also confirmed the biological role of YAP in AS. YAP interacted with SMAD7, and YAP up-regulation inhibited the process of EndMT in ox-LDL-treated HUVECs. YAP overexpression-mediated inhibition of EndMT in ox-LDL-treated HUVECs was reversed by up-regulation of miR-200c-3p. Thus, miR-200c-3p overexpression accelerated EndMT in ox-LDL-treated HUVECs by suppressing YAP expression. Nevertheless, this work only determined the functional role of miR-200c-3p on EndMT of HUVECs. Whether miR-200c-3p affects proliferation and migration properties of HUVECs is still unknown. We will conduct research on this topic in further work.

## Conclusions

In conclusion, this work demonstrated that miR-200c-3p accelerated EndMT in ox-LDL-treated HUVECs by suppressing SMAD7/YAP pathway. Thus, miR-200c-3p may be an important molecule for the onset of atherosclerosis.

### Supplementary Information

**Additional file 1: Figure S1**. SMAD7 and YAP were down-regulated in ApoE^−/−^ mice and ox-LDL-treated HUVECs. ApoE^−/−^ mice were fed with HFD to establish AS mouse model. Normally fed C57B/6 mice served as control. The mRNA and protein expression of SMAD7 and YAP in aortic tissues of C57B/6 and ApoE^−/−^ mice was assessed by qRT-PCR (A) and WB analysis (B-C). HUVECs were treated with ox-LDL at different concentrations (0, 10, 20, 40 μg/mL) for 48 h. The mRNA and protein expression of SMAD7 and YAP in HUVECs was assessed by qRT-PCR (D) and WB analysis (E–F). **P < 0.01 vs. C57B/6 mice group. ^#^*P* < 0.05, ^##^*P* < 0.01, ^###^*P* < 0.001 vs. 0 group.

**Additional file 2: Figure S2.** The proportions of spindle-shaped mesenchymal cells in ox-LDL-treated HUVECs. (A) HUVECs were transfected with miR-200c-3p mimic or mimic NC, followed by 40 μg/mL ox-LDL treatment. (B) HUVECs were co-transfected with miR-200c-3p mimic or mimic NC and pcDNA3.1-SMAD7 or pcDNA3.1-NC, followed by 40 μg/mL ox-LDL treatment. (C) HUVECs were co-transfected with miR-200c-3p mimic or mimic NC and pcDNA3.1-YAP or pcDNA3.1-NC, followed by 40 μg/mL ox-LDL treatment. Quantitative analysis of proportions of spindle-shaped mesenchymal cells in HUVECs with Rhodamine phalloidin staining. **P < 0.01 vs. ox-LDL + mimic NC or mimic NC + vector group. ^##^*P* < 0.01 vs. Control, mimic NC + SMAD7 or mimic NC + YAP group.

## Data Availability

The datasets used and/or analyzed during the current study are available from the corresponding author on reasonable request.
