# Supplementary figures and images for: MiR-200c-3p promotes ox-LDL-induced endothelial to mesenchymal transition in human umbilical vein endothelial cells through SMAD7/YAP pathway
Source: J Physiol Sci. 2021 Sep 15;71:30. doi: 10.1186/s12576-021-00815-z (PMC10717414; doi:10.1186/s12576-021-00815-z)

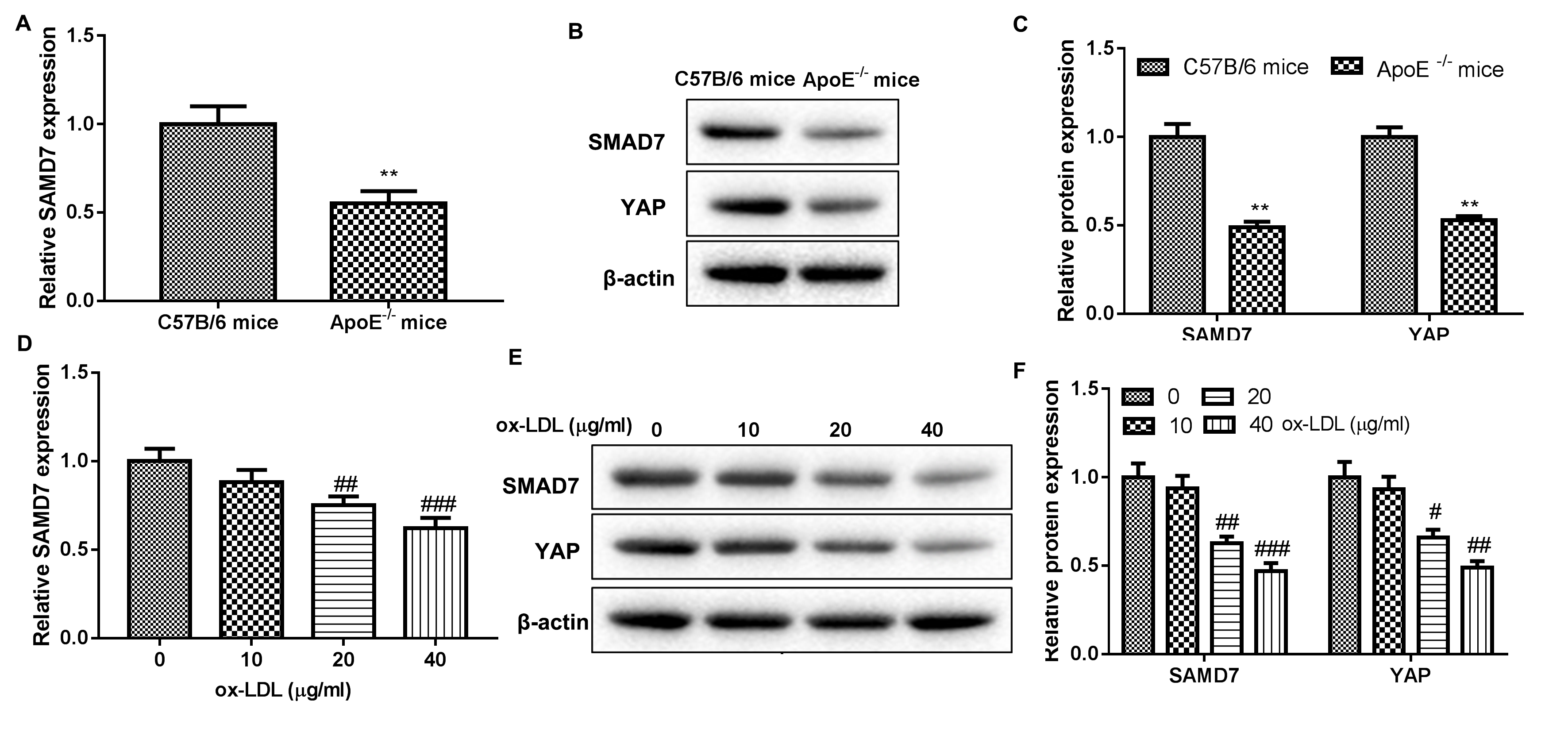

Supplement: Supplementary file 1 — Additional file 1: Figure S1. SMAD7 and YAP were down-regulated in ApoE−/− mice and ox-LDL-treated HUVECs. ApoE−/− mice were fed with HFD to establish AS mouse model. Normally fed C57B/6 mice served as control. The mRNA and protein expression of SMAD7 and YAP in aortic tissues of C57B/6 and ApoE−/− mice was assessed by qRT-PCR (A) and WB analysis (B-C). HUVECs were treated with ox-LDL at different concentrations (0, 10, 20, 40 μg/mL) for 48 h. The mRNA and protein expression of SMAD7 and YAP in HUVECs was assessed by qRT-PCR (D) and WB analysis (E–F). **P < 0.01 vs. C57B/6 mice group. #P < 0.05, ##P < 0.01, ###P < 0.001 vs. 0 group. [file 12576_2021_815_MOESM1_ESM.tif]

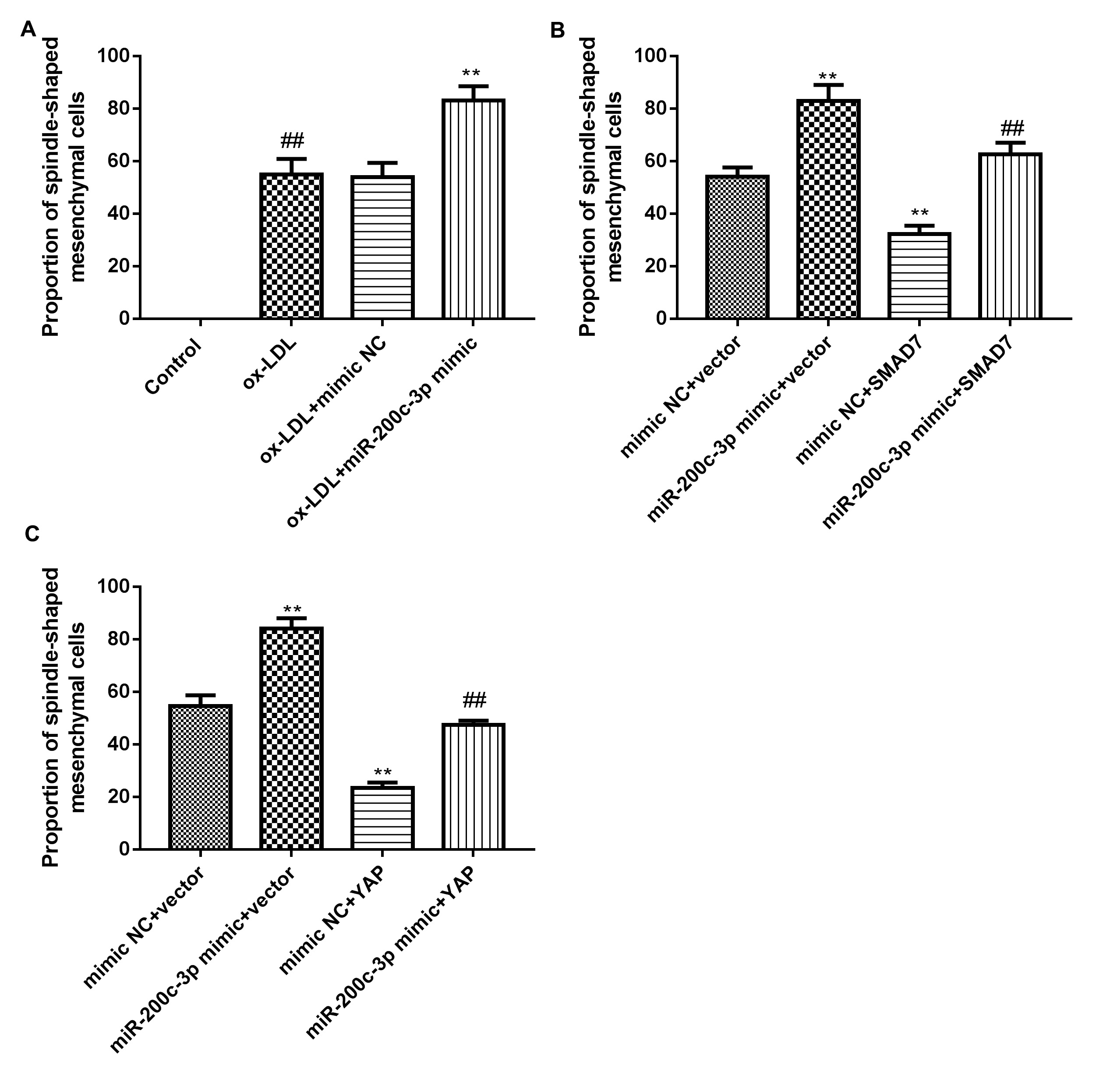

Supplement: Supplementary file 2 — Additional file 2: Figure S2. The proportions of spindle-shaped mesenchymal cells in ox-LDL-treated HUVECs. (A) HUVECs were transfected with miR-200c-3p mimic or mimic NC, followed by 40 μg/mL ox-LDL treatment. (B) HUVECs were co-transfected with miR-200c-3p mimic or mimic NC and pcDNA3.1-SMAD7 or pcDNA3.1-NC, followed by 40 μg/mL ox-LDL treatment. (C) HUVECs were co-transfected with miR-200c-3p mimic or mimic NC and pcDNA3.1-YAP or pcDNA3.1-NC, followed by 40 μg/mL ox-LDL treatment. Quantitative analysis of proportions of spindle-shaped mesenchymal cells in HUVECs with Rhodamine phalloidin staining. **P < 0.01 vs. ox-LDL + mimic NC or mimic NC + vector group. ##P < 0.01 vs. Control, mimic NC + SMAD7 or mimic NC + YAP group. [file 12576_2021_815_MOESM2_ESM.tif]
